# Expression of the γ-phosphorylated histone H2AX in gastric carcinoma and gastric precancerous lesions

**DOI:** 10.3892/ol.2015.2896

**Published:** 2015-01-26

**Authors:** ZHONG GUO, SHUYAN PEI, TIANBIN SI, JING LI, CHAO JIANG, SHANGBIAO LI, JIN ZHAO

**Affiliations:** 1 Laboratory of Functional Science, Medical College of Northwest University for Nationalities, Lanzhou, Gansu 730030, P.R. China; 2 Department of Gynecologic Oncology, Gansu Cancer Hospital, Lanzhou, Gansu 730050, P.R. China

**Keywords:** γH2AX, superficial gastritis, atrophic gastritis, gastric carcinoma

## Abstract

The histone γH2AX is a marker of activated DNA damage that is overexpressed in various cancers and corresponding precursor lesions, indicating that γH2AX is a component in oncogenic transformation. The present study aimed to determine whether the immunohistochemical expression of γH2AX is involved in the progression between superficial gastritis (n=20), atrophic gastritis (n=24) and gastric carcinoma (n=79). There was no increase in γH2AX expression between superficial and atrophic gastritis, but there was a significant increase in γH2AX expression between these two conditions and gastric carcinoma (χ^2^=68.712; P<0.001). The expression of γH2AX in moderately-differentiated gastric adenocarcinoma (n=49) was evidently higher compared with poorly-differentiated gastric adenocarcinoma (n=26; χ^2^=14.241; P<0.01). Staining for γH2AX did not reveal a significant association between the expression of the histone and patient age, depth of invasion, lymph node metastasis or the tumor-node-metastasis stage of the gastric carcinoma. Overall, the present study demonstrated that enhanced γH2AX expression may be closely associated with gastric carcinoma, but is less likely to be involved in the genesis of gastric carcinoma.

## Introduction

As an evolutionary process that selects for genetic and epigenetic changes, tumorigenesis allows for the evasion of anti-proliferative and cell death-inducing mechanisms, which normally limit the clonal expansion of somatic cells (1). The majority of tumors develop genetic instability, but it is unclear how early this occurs and if this drives tumor development (2). One of the mechanisms of tumor suppression is the DNA damage response (DRR), which is activated in the early stages of human tumorigenesis and leads to cell-cycle blockade or apoptosis, thereby constraining tumor progression (3,4).

DNA double-strand breaks (DSBs) can arise from errors that occur during DNA replication, from external agents, including ionizing radiation, or during genomic rearrangements. DSBs induce chromosomal aberrations that cause cells to malfunction, resulting in cell death or tumorigenesis (5). One of the earliest steps in the cellular response to DSBs is the phosphorylation of histone H2AX at serine 139, the site of γ-phosphorylation, resulting in γH2AX (6). H2AX is phosphorylated by phosphoinositide-3 (PI3) kinases, including ataxia telangiectasia mutated (ATM), DNA-dependent protein kinase and ataxia telangiectasia and Rad3 related. The number of γH2AX foci is a notable marker for DSBs. In addition to γH2AX, activated PI3 kinases also initiate other downstream events, including the phosphorylation of BRCA1, Chk2 and p53 (7,8). Immunohistochemical analyses of γH2AX have been performed in human cancers of the bladder, breast, lung, colon and prostate (4,9,10). γH2AX-positive cells have been reported to be present in colorectal cancer (CRC) and precursor lesions, including adenoma, but not in the normal colonic epithelium (11). Furthermore, tissue from invasive CRC has been reported to exhibit decreased staining for γH2AX compared with adenoma tissue (4). These results indicate that staining of γH2AX is associated with DNA damage checkpoint activation in premalignant lesions. Therefore, the existence of γH2AX foci may be a useful and sensitive marker of cancer, particularly for detecting cancers or precursor lesions.

Worldwide, gastric cancer is the second leading cause of cancer-associated mortality. The majority of gastric cancers are diagnosed at a late stage, leading to a five-year survival rate of ≤25% (12). This late-stage diagnosis and the resulting low survival rate, in addition to the majority of gastric cancers being preceded by clearly recognizable precursors, has led to numerous studies investigating the possibility of screening and surveillance (13). The precursor lesions of gastric cancer are well-characterized and consist of atrophic gastritis, intestinal metaplasia or low-grade dysplasia (14). However, to the best of our knowledge, the expression of γH2AX in gastric cancer and its precursor lesions has not been investigated at present. The present study investigated the effect of γH2AX expression on the progression of the morphological spectrum between superficial and atrophic gastritis and gastric cancer.

## Materials and methods

### Patients and tumor specimens

For the present immunohistochemical analysis, formalin-fixed, paraffin-embedded archival tissues were used. The tissues were obtained from 123 patients that were diagnosed with superficial gastritis (n=20), atrophic gastritis (n=24) or gastric carcinoma (n=79). The patients diagnosed with gastric carcinoma consisted of 49 patients with moderately-differentiated gastric adenocarcinoma, 26 patients with poorly-differentiated adenocarcinoma, one patient with signet ring cell cancer, one patient with salivary mucoepidermoid carcinoma and two patients with mucoid carcinoma. All patients underwent gastroscopy and surgery between 2011 and 2012 at Gansu Cancer Hospital (Lanzhou, Gansu, China). There was no significant difference in the age or gender distribution between these groups. For the gastric cancer patients, only patients who did not undergo pre-operative radio- or chemotherapy were enrolled in the present study. Pathological diagnosis and classification were performed according to the criteria of the World Health Organization (9,10). All specimens were collected under the approval of the Ethics Comittee of The Medical College of Northwest University for Nationalities (Lanzhou, China) with consent from the patients.

### Immunohistochemical analysis of γH2AX

One representative tumor block from each patient, including the tumor center, invasive front and tumor-associated non-neoplastic mucosa, was examined by immunohistochemistry. In cases of large, late-stage tumors, various sections were examined to include representative areas of the tumor center and lateral and deep invasive fronts.

The paraffin-embedded tissues that were used for the original hematoxylin and eosin-stained sections were also chosen for immunohistochemistry. Sections of paraffin-embedded tumor tissue (5 μm thick) were prepared, and subsequent to dewaxing and rehydrating the tissue using graded ethanol series, the slides were immersed in high-pH target retrieval solution (Dako, Glostrup, Denmark) in a 95°C water bath for 30 min. The slides were washed in Tris-buffered saline, blocked for 10 min, and incubated with polyclonal goat anti-mouse γH2AX antibody (1:500 dilution; cat. no. 115-545-003, Jackson ImmunoResearch Laboratories, Inc., West Grove, PA, USA) for 2 h. The slides were rinsed and incubated for 1 h with fluorescein isothiocyanate-conjugated anti-mouse goat F(ab′)^2^ fragment (Dako). To stain the nuclei, the slides were submersed in 0.05 μg/ml DAPI for 5 min, rinsed and mounted using 10 μl fluorescent mounting media (KPL, Gaithersburg, MD, USA). The tumor sections were observed using an Olympus BX53 fluorescent microscope (Olympus, Tokyo, Japan). As this was considered a feasibility study, no specific procedures were adopted to ensure that images were obtained randomly across the section. As sections rather than whole cells were scored, cells possessing one or more γH2AX foci were counted as positive. Efforts were made to score only tumor regions, and evident regions of necrosis or stroma were not included in the analysis. The foci results were presented as averages of the scores for several high-power images. In total, 8–12 digitized images, containing 50–100 nuclei each, were scored by eye for the percentage of nuclei presenting γH2AX foci, and the results were presented as averages. Finally, the sections were scored semi-quantitatively as follows: (1+), <10% immunopositive cells; (2+), 10–50% immunopositive cells; and (3+), >50% immunopositive cells (15–17).

### Statistical methods

Associations between the clinicopathological variables and immunostaining for γH2AX were analyzed using a χ^2^-test. SPSS 17.0 software (SPSS, Inc., Chicago, IL, USA) was used for the statistical analysis. P<0.05 was considered to indicate a statistically significant difference.

## Results

**Figure 1:**
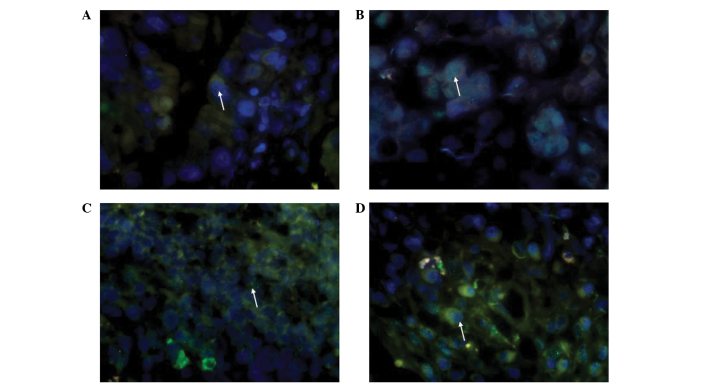
Immunohistochemical analysis of γH2AX in (A) superficial gastritis, (B) atrophic gastritis, (C) moderated differentiated gastric adenocarcinoma and (D) poorly-differentiated gastric adenocarcinoma. Granular staining of γH2AX is found in the nucleus (magnification, ×400).

High-power images of these biopsies were analyzed visually to determine the fraction of nuclei that were γH2AX-positive, which was defined as cells possessing one or more foci per nucleus. γH2AX produced granular nuclear staining. It was confirmed that γH2AX demonstrated ubiquitous staining in gastric adenocarcinoma, particularly in moderately-differentiated gastric adenocarcinoma, and that superficial and atrophic gastritis demonstrate only partial staining (Fig. 1A–D). It has previously been reported that γH2AX is expressed during early apoptosis triggered through the caspase 3/caspase-activated DNase pathway (18) and that γH2AX-positive tumor cells in superficial portions or necrotic debris were also positive for the activated form of caspase-3, which is a marker of apoptosis. Therefore, cases with superficial staining and staining of necrotic debris were not classified as positive for γH2AX expression in the present study.

Gastric carcinoma tissue expresses a higher level of γH2AX compared with superficial and atrophic gastritis tissue (Table I; χ^2^=68.712; P<0.001). On average, 34.43% nuclei in gastric carcinoma tissues possess one or more γH2AX foci, ranging between 5.34 and 72.71%. However, in superficial and atrophic gastritis, only 6.35 and 9.75% of nuclei, respectively, contained foci. Poorly-differentiated gastric adenocarcinoma expressed lower levels of γH2AX compared with moderately-differentiated adenocarcinoma (Table II; χ^2^=14.241; P<0.01). In poorly-differentiated gastric adenocarcinoma, 26.55% of nuclei contained one or more γH2AX foci, ranging between 5.64 and 66.71%, and in moderately-differentiated adenocarcinoma, 39.31% of nuclei contained one or more γH2AX foci, ranging between 5.75 and 72.73%.

The association between γH2AX staining and clinicopathological parameters in gastric adenocarcinoma was also analyzed. γH2AX staining demonstrated no significant association with age, depth of invasion, lymph node metastasis or the tumor-node-metastasis (TNM) stage in gastric carcinoma (Table III).

## Discussion

In previous years, evidence has emerged to support the hypothesis that there is aberrant activation of the DDR checkpoint in human epithelial pre-cancerous lesions. Bartek *et al* (11) reported extensive abnormalities of the ATM-Chk2 axis in non-invasive precursor lesions of bladder, colon and breast cancers. By contrast, Gorgoulis *et al* (9) reported similar results obtained from lung and epidermal tissues. In all these instances, DDR checkpoint activation was accompanied by evidence of DNA double-strand breaks, as assessed by γH2AX expression. In addition, DDR has been observed in pre-cancerous lesions prior to the onset of the genomic instability that characterizes invasive cancer, indicating that widespread allelic imbalances were not the underlying basis for checkpoint activation within the epithelium (19).

While DDR is a major component in tumor suppression, the mechanism behind this response actively participating in the suppression of gastric tumorigenesis remains elusive (20). In the present study, the expression of γH2AX in gastric tissues was investigated and a significant immunoexpression was noted and confirmed by statistical evaluation.

The current study provided evidence for the presence of a DDR in gastric cancer tissue, which is characterized by staining for γH2AX. The γH2AX levels, which were assessed by nuclear staining scores, were significantly higher in gastric cancer tissues compared with superficial and atrophic gastritis tissues. Certain cells exhibited a notable increase in nuclear staining for γH2AX and others demonstrated distinct nuclear foci, which were confirmed by fluorescence microscopy. The majority of tumors develop genetic instability, but the rapidity with which this occurs and the ability to drive tumor development remains unclear (21). From the initial changes, cancer development is associated with DNA replication stress, leading to DNA double-strand breaks, genomic instability and selective pressure for p53 mutations. Several mechanisms to constrain oncogenesis have been proposed, including hypoxia, telomere attrition and reactive oxygen species (22).

Secondly, the present study revealed that the expression of γH2AX differs between poorly- and moderately-differentiated gastric adenocarcinoma. Moderately-differentiated gastric adenocarcinoma contained a higher number of γH2AX foci compared with poorly-differentiated gastric adenocarcinoma, and the expression levels of γH2AX were independent of the depth of invasion and TNM stage in the two gastric adenocarcinoma types. It was hypothesized that in the poorly-differentiation gastric adenocarcinoma tissues a serious DDC had occurred, resulting in the lower levels of γH2AX. When the DNA damage in cells is too serious to be repaired, γH2AX-positive cells undergo apoptosis. An additional explanation is that the molecular mechanisms underlying the phosphorylation of γH2AX may differ between moderately- and poorly-differentiated gastric adenocarcinoma tissues. It is also possible that, due to a single DSB being able to result in chromosomal translocations, deletions or loss of genetic information, several genes associated with tumor progression may be deleted in H2AX-positive gastric carcinoma tissues (17).

In the present study, the mechanism behind the decreased expression of γH2AX in atrophic gastritis remains to be elucidated. γH2AX has been reported to be commonly expressed in early precursor lesions located in the bladder, breast, lung, colon and prostate (9,10). Atrophic gastritis is considered as a pre-malignant gastric lesion in patients at risk of progression to gastric cancer (23). It was therefore hypothesized that atrophic gastritis should demonstrate a higher DDR. Certain studies support the present result (24–26). In non-neoplastic gastric mucosa or intestinal metaplasia adjacent to the tumor, only a few superficial cells demonstrated immunostaining of γH2AX. Furthermore, in intestinal metaplasia adjacent to the tumor, which is considered to be a gastric precancerous lesion, staining of γH2AX was not observed in epithelial or stromal cells (14). In addition, a previous large cohort study revealed that the risk of progression to cancer within 10 years was only 0.8% for individuals with atrophic gastritis (12). These results suggest that DSBs are less likely to be involved in the genesis of gastric carcinoma.

Although the number of cases examined in the present study was relatively small, it was revealed that γH2AX is overexpressed in gastric carcinoma, and that the expression of γH2AX is higher in moderately-differentiated gastric adenocarcinoma compared with poorly-differentiated gastric adenocarcinoma. These results demonstrated that enhanced γH2AX expression may be closely associated with gastric carcinoma, but is less likely to be involved in the genesis of gastric carcinoma. However, additional details require further investigation.

## Figures and Tables

**Table I:** Immunohistochemical analysis of γH2AX expression in superficial gastritis, atrophic gastritis and gastric carcinoma.

		γ-H2AX expression, n				
				
Diagnosis	n	+	++	+++	χ^2^	P-value
Superficial gastritis	20	19	1	0		
Atrophic gastritis	26	21	5	0	68.712	0.000
Gastric carcinoma	79	10	49	20		

**Table II:** Immunohistochemical analysis of the difference in γH2AX expression between moderately- and poorly-differentiated gastric adenocarcinoma.

		γH2AX expression, n				
				
Gastric adenocarcinoma	n	+	++	+++	χ^2^	P-value
Moderately-differentiated	49	1	34	14	14.241	0.007
Poorly-differentiated	26	8	13	5		

**Table III:** Correlation between γH2AX expression and the clinicopathological features of gastric carcinoma.

		γH2AX expression, n				
				
Clinicopathological features	n	+	++	+++	χ^2^	P-value
Age, years						
<55	28	6	16	6	3.019	0.221
≥55	51	4	34	13		
Depth of invasion						
Tis-1	11	3	7	1	3.346	0.188
T2–4	68	7	43	18		
Lymph node metastasis						
Present	69	10	43	16	1.699	0.221
Absent	10	0	7	3		
Distant metastasis						
Present	30	3	19	8	0.117	0.943
Absent	49	6	31	12		
TNM stage						
0–I	11	1	9	1	1.838	0.399
II–IV	68	8	42	18		

TNM, tumor-node-metastasis.

## References

[1] Merlo LM, Pepper JW, Reid BJ, Maley CC (2006). Cancer as an evolutionary and ecological process. Nat Rev Cancer.

[2] Bodmer W, Bielas JH, Beckman RA (2008). Genetic instability is not a requirement for tumor development. Cancer Res.

[3] Woo RA, McLure KG, Lees-Miller SP, Rancourt DE, Lee PW (1998). DNA-dependent protein kinase acts upstream of p53 in response to DNA damage. Nature.

[4] Bartkova J, Horejsí Z, Koed K (2005). DNA damage response as a candidate anti-cancer barrier in early human tumorigenesis. Nature.

[5] van Gent DC, Hoeijmakers JH, Kanaar R (2001). Chromosomal stability and the DNA double-stranded break connection. Nat Rev Genet.

[6] Rogakou EP, Pilch DR, Orr AH, Ivanova VS, Bonner WM (1998). DNA double-stranded breaks induce histone H2AX phosphorylation on serine 139. J Biol Chem.

[7] Paull TT, Rogakou EP, Yamazaki V (2000). A critical role for histone H2AX in recruitment of repair factors to nuclear foci after DNA damage. Curr Biol.

[8] Zhao J, Guo Z, Zhang H (2013). The potential value of the neutral comet assay and γH2AX foci assay in assessing the radiosensitivity of carbon beam in human tumor cell lines. Radiol Oncol.

[9] Gorgoulis VG, Vassiliou LV, Karakaidos P (2005). Activation of the DNA damage checkpoint and genomic instability in human precancerous lesions. Nature.

[10] Fan C, Quan R, Feng X (2006). ATM activation is accompanied with earlier stages of prostate tumorigenesis. Biochim Biophys Acta.

[11] Spruck CH, Won KA, Reed SI (1999). Deregulated cyclin E induces chromosome instability. Nature.

[12] den Hoed CM, Holster IL, Capelle LG (2013). Follow-up of premalignant lesions in patients at risk for progression to gastric cancer. Endoscopy.

[13] Van Cutsem E, Dicato M, Geva R (2011). The diagnosis and management of gastric cancer: expert discussion and recommendations from the 12th ESMO/World Congress on Gastrointestinal Cancer, Barcelona, 2010. Ann Oncol.

[14] Karimi P, Islami F, Anandasabapathy S, Freedman ND, Kamangar F (2014). Gastric cancer: descriptive epidemiology, risk factors, screening, and prevention. Cancer Epidemiol Biomarkers Prev.

[15] Sentani K, Oue N, Sakamoto N (2008). Positive immunohistochemical staining of gammaH2AX is associated with tumor progression in gastric cancers from radiation-exposed patients. Oncol Rep.

[16] Olive PL, Banuelos CA, Durand RE, Kim JY, Aquino-Parsons C (2010). Endogenous and radiation-induced expression of gammaH2AX in biopsies from patients treated for carcinoma of the uterine cervix. Radiother Oncol.

[17] Brustmann H, Hinterholzer S, Brunner A (2011). Expression of phosphorylated histone H2AX (γ-H2AX) in normal and neoplastic squamous epithelia of the uterine cervix: an immunohistochemical study with epidermal growth factor receptor. J Gynecol Pathol.

[18] Lu C, Zhu F, Cho YY (2006). Cell apoptosis: requirement of H2AX in DNA ladder formation, but not for the activation of caspase-3. Mol Cell.

[19] Simão ÉM, Sinigaglia M, Bugs CA, Castro MA, Librelotto GR, Alves R, Mombach JC (2012). Induced genome maintenance pathways in pre-cancer tissues describe an anti-cancer barrier in tumor development. Mol Biosyst.

[20] Ohnishi S, Ma N, Thanan R, Pinlaor S, Hammam O, Murata M, Kawanishi S (2013). DNA damage in inflammation-related carcinogenesis and cancer stem cells. Oxid Med Cell Longev.

[21] Bartek J, Bartkova J, Lukas J (1997). The retinoblastoma protein pathway in cell cycle control and cancer. Exp Cell Res.

[22] Olive PL (2011). Retention of γH2AX foci as an indication of lethal DNA damage. Radiother Oncol.

[23] Feng XS, Wang YF, Hao SG (2013). Expression of Das-1, Ki67 and sulfuric proteins in gastric cardia adenocarcinoma and intestinal metaplasia lesions. Exp Ther Med.

[24] Ock CY, Kim EH, Choi DJ, Lee HJ, Hahm KB, Chung MH (2012). 8-Hydroxydeoxyguanosine: not mere biomarker for oxidative stress, but remedy for oxidative stress-implicated gastrointestinal diseases. World J Gastroenterol.

[25] Hardbower DM, Peek RM, Wilson KT (2014). At the Bench: Helicobacter pylori, dysregulated host responses, DNA damage, and gastric cancer. J Leukoc Biol.

[26] Kawanishi S, Hiraku Y (2006). Oxidative and nitrative DNA damage as biomarker for carcinogenesis with special reference to inflammation. Antioxid Redox Signal.

